# The Use of Multidimensional Image-Based Analysis to Accurately Monitor Cell Growth in 3D Bioreactor Culture

**DOI:** 10.1371/journal.pone.0026104

**Published:** 2011-10-20

**Authors:** Marc-Olivier Baradez, Damian Marshall

**Affiliations:** Science and Technology Division, LGC, Teddington, Middlesex, United Kingdom; University of São Paulo, Brazil

## Abstract

The transition from traditional culture methods towards bioreactor based bioprocessing to produce cells in commercially viable quantities for cell therapy applications requires the development of robust methods to ensure the quality of the cells produced. Standard methods for measuring cell quality parameters such as viability provide only limited information making process monitoring and optimisation difficult. Here we describe a 3D image-based approach to develop cell distribution maps which can be used to simultaneously measure the number, confluency and morphology of cells attached to microcarriers in a stirred tank bioreactor. The accuracy of the cell distribution measurements is validated using *in silico* modelling of synthetic image datasets and is shown to have an accuracy >90%. Using the cell distribution mapping process and principal component analysis we show how cell growth can be quantitatively monitored over a 13 day bioreactor culture period and how changes to manufacture processes such as initial cell seeding density can significantly influence cell morphology and the rate at which cells are produced. Taken together, these results demonstrate how image-based analysis can be incorporated in cell quality control processes facilitating the transition towards bioreactor based manufacture for clinical grade cells.

## Introduction

The use of living cells in clinical applications offers great benefits over traditional treatments potentially allowing damaged and diseased tissues to be repaired rather than replaced. However, producing cells in the quantities required for cell based therapies presents many challenges, particularly as the cells often have to be adhered to a substrate, limiting the numbers of cells that can be produced using standard cell culture practices. This is driving the need for the development of new culture processes which not only have the robustness of traditional methods but are also efficient and scalable enough to produce cells in the amounts required for therapeutic application [1].

A promising approach for producing large numbers of cells is the use of bioreactors. These systems have been used extensively within the bioprocessing industry for many years to grow suspension cells for the manufacture of high value biochemicals (e.g. antibody production by hybridoma cells) [2] but are now increasingly being applied for the production of cells which require anchorage to a substrate in order to grow. One of the most commonly applied approaches is to use cells adhered to the surface of three dimensional (3D) microcarriers in a stirred tank bioreactor [3]. This approach provides a large surface area for cell production, due to the surface area of the microcarriers, while the stirring provides a homogenous culture environment, facilitating mass transfer of nutrients to all cells [4] thereby achieving higher cell yields than conventional (2D) culture methods. Scaling production of cells, using different microcarrier systems in stirred tank bioreactors, has been shown, under optimal conditions, to increase the yield of cells by as much as 12 fold when compared with traditional culture methods [5] and has been applied to a range of cell therapy models including mesenchymal stem cells [6], [7], embryonic stem cells [5], [8], fibroblasts [9] and keratinocytes [10]. Despite these proof of concept reports, bioreactor based cell production is still mostly performed at the pilot scale (up to 1 litre volume) and in-process monitoring of the cells is usually limited.

Measuring cell growth and assessing cell quality in standard culture is usually achieved using simple imaging techniques such as brightfield microscopy which can be used to monitor several parameters simultaneously. Cell morphology, viability and proliferation, which are good indicators of cell health, can be monitored *pro re nata* to ensure quality, while cell number and confluency (the percentage of the growth surface covered by cells) can be used to judge the optimal point at which to retrieve cells from culture in order to maximise cell yields. In bioreactor cultures these multiparametric measurements are more complicated due to the fact that the cells are adhered onto a 3D growth substrate and as such most reports on the growth of cells in bioreactors rely on a single measure of cell number using either direct or indirect measurements (Table 1). Direct measurements [8], [11]–[26] require the cells to be removed enzymatically from the growth substrate and stained using cell viability dyes for bright field (trypan blue exclusion assay) or fluorescence microscopy (live/dead assays, Hoechst for nuclear labelling). These methods provide the most quantitative results, but the requirement for cells to be detached from the substrate affects both cell number and viability and means that important information about cell confluency and morphology are lost. Indirect monitoring techniques [2], 3,8,12,13,23–33 do not require the cells to be removed from the growth substrate and instead estimate cell growth based on parameters such as the depletion of nutrients by the cells from the culture medium (an indication of cell metabolism rates) or cell number based on the enzymatic metabolism of compounds within the cells. For example, the MTT assay which estimates cell number based on the reduction of tetrazolium salts to formazan in the mitochondria. These methods, while easier to perform, are less sensitive than direct methods and provide no information on cell quality characteristics such as morphology and confluency.

**Table 1:** Measurement techniques used to monitor the quality of adherent cells in bioreactor culture.

	Measurement Technique	Culture system	References
**Direct**	Trypan Blue	Microcarriers	8, 11, 13–15, 17–19
*Visualisation*		Aggregates	12, 16, 20
	Fluorescent markers	Microcarriers	17, 21
		3D Scaffold	22
	Nuclear counting	Microcarriers	8, 13, 21–24
		Aggregates	24
	Light/Electron microscopy	Microcarriers	17
		Aggregates	25
	Alamar Blue	Microcarriers	26
	Flow cytometry	Microcarriers	17
**Indirect**	Nutrient/Metabolites	Microcarriers	8, 13, 23–28, 31
*Metabolism*		Aggregates	2, 12, 28–30
		Silk Scaffold	32
	Cytosolic enzymes	Microcarriers	3, 23–26
		Aggregates	12, 24
	MTT	Microcarriers	13, 26, 33
	Total DNA	Aggregates	12
		Silk Scaffold	32

Ideally, a system is required for direct measurement of cell number and viability in cells that remain attached to the 3D growth substrate so that these measurements can be combined with information on cell morphology and confluency, allowing multiparametric analysis. To make these measurements quantitative, this would require a system which allows the simultaneous measurement of thousands of individual cells attached to hundreds of microcarrier beads. An approach which could be used to generate this information is 3D imaging, although the lack of speed with which the image data sets can be acquired, processed and analysed in the 3D volume may be prohibitive. In this paper we investigate a novel approach to processing 3D image data to create 2D cell distribution maps which can be used for rapid direct analysis of the number, confluency and morphology of cells adhered to the surface of microcarrier beads. This approach which is validated using *in silico* modelling is applied to an exemplar model system of human dermal fibroblast cells grown in a stirred tank bioreactor to demonstrate its use for monitoring proliferation and the health of cells under different manufacture conditions.

## Materials and Methods

### Standard cell culture

Cultures of Human dermal fibroblasts (HDF) (LGC Standards, UK) were prepared by growing the cells adhered to T-175 cell culture flasks (Corning, UK) in Dulbecco's Modified Eagle Medium containing 10% fetal bovine serum (DMEM), buffered at pH 7.4 using 30 mM HEPES. Cells were maintained at 37°C in a humidified atmosphere with media changes every 48–72 hours until ready for use. Cells from passage 12 to 17 were used for all experiments.

### Bioreactor cultures

Approximately 6.45×10^6^ Cytodex 1 microcarrier beads (GE Healthcare, UK) were prepared by washing 3 times in phosphate buffered saline (PBS) solution for 4 h and sterilised in 70% ethanol overnight. The beads were rinsed 3 times in PBS, re-suspended in DMEM and placed into a 125 ml glass stirred tank bioreactor (Corning, UK). HDF cells were enzymatically dissociated from their culture flasks by incubating the cells for 5 min with 0.025% Trypsin-EDTA and resuspended in an equal volume of DMEM. Cell number and viability were measured using the trypan-blue assay. The HDF cells were seeded onto microcarriers by mixing cells and microcarriers at a ratio of 5∶1 or 10∶1 in the stirred tank bioreactor. Cells were allowed to attach to the microcarriers for 24 hours with 1 min 45 sec intermittent stirring at 40 RPM every 45 min. After 24 hours the stirring regime was changed to 2 min intermittent stirring every 20 min for the remainder of the culture process. DMEM growth media was replenished every 48–72 hours by replacing 60% of the media.

### Fluorescent labelling of live and dead cells

5 ml samples of DMEM media containing cells and microcarriers were removed from the stirred tank bioreactors under sterile conditions at specific time points over a period of 13 days. The microcarriers in each sample were allowed to settle by gravity. Once settlement was complete the supernatant was removed and the microcarriers were re-suspended in 1 ml of fresh DMEM culture medium containing 4 µM calcein-AM (to label live cells) and 2 µM ethidium homodimer-1 (to label dead cells) (Invitrogen, UK) and incubated at 37°C for 30 min.

### Laser scanning confocal microscopy

Fluorescently labelled cell samples were imaged using a Nikon Eclipse TE2000 inverted laser scanning confocal microscope. Images were acquired using the EZ-C1 acquisition and operating software. Low magnification images were acquired at ×10 magnification. A minimum of 10 fields of view comprising 15–30 microcarriers each were imaged for each time point. Z-interval was set to a constant increment of 2.5 µm between successive focal planes. An average of 100 images was collected per field of view, encompassing the entire microcarrier volume. Live cells were imaged at 488 nm excitation wavelength, dead cells at 543 nm. Laser power was kept constant between each imaging session.

### Development of cell distribution maps

Confocal images were processed using Matlab (R2008b, The MathWorks, USA) for automatic detection of microcarriers, mapping of live cell distribution around microcarriers, and quantification of confluence level. For each dataset, a Hough transform was applied to the top maximum projection image to identify microcarrier positions and radii in the 2D *X-Y* plane. A sub-volume encompassing each microcarrier was extracted and further processed along the *Z*-axis in order to identify top and bottom coordinates of the microcarrier. A grid with resolution finer than the original images was mapped onto the surface of a virtual sphere centred on the microcarrier to prevent information loss from sub-optimal sampling. Fluorescence intensity was iteratively sampled 30 times along the coordinates of this spherical grid in the vicinity of the microcarrier surface, measuring fluorescence along orthogonal lines to the microcarrier surface, below, at and above this surface. The process generated a 500×500×30 matrix *M_x,y,z_* (surface = 500×500 points *x-y*, depth = 30 points *z*) which was further processed in order to obtain a final map of cell distribution, defined as
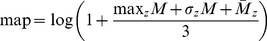
(1)where 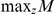 = maximum intensity value, 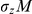 = standard deviation, and 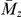 = mean intensity, all computed along the *z* dimension of *M* and normalised to their maximum value. The logarithm was used to transform the intensity distribution so that background values were Normally distributed. In order to compensate for batch-to-batch variability, all maps obtained in a given field of view were normalised by scaling their values in the range [0, 1].

### Cell confluency algorithm

For all cell distribution maps from one Z-stack, symmetrical 2D intensity co-occurence histograms *C*(Δ*x*, Δ*y*) were computed using pairs of points separated by Δ*x* = 10 pixels (Δ*y* = 0). The distance Δ*x* was chosen so the likelihood of co-occurrence of background pairs of pixels (from uniform areas) was significantly increased compared to the more variable cell signals. Median values were computed down the columns of *C* and all profiles from the same batch (i.e. Z-stack) were averaged to smooth low frequency cell signal co-occurrences whilst emphasizing the distribution of background noise co-occurrences. The resulting profile *P_C_* was smoothed with a 5×1 averaging kernel, and its absolute first derivative computed as Δ*P_c_*. This curve had high magnitude in regions of large fluctuations of *P_c_* (allegedly background) and low magnitude elsewhere (in the largest proportion of the dynamic range representing cell signal). The threshold *T* for separating background from signal values was defined as the last intensity for which Δ*P_c_* was higher than the value mean(Δ*P_c_*)+1.96×SD(Δ*P_c_*). The final confluence measurement was calculated as the percentage of signal area in a map, corrected for spherical distortion induced by mapping the 2D surface of a 3D spherical microcarrier onto a 2D plane.

### Validation of confluence measurements

Artificial *in silico* microcarriers and cells were modelled to quantify the performance of the segmentation algorithm, estimate the true confluence of the cells in 3D, and assign an error to the estimated true confluence. Artificial maps were created by random addition of *N* 2D Gaussian kernels of various sizes and magnitudes:
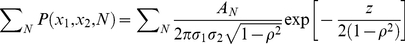
(2)where 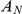 is the magnitude of the *N* ^th^ kernel, the variables 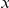 are spatial coordinates along dimensions 1 and 2, 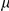 are positions of the kernel peaks, 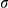 are their standard deviations,
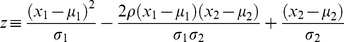
(3)and
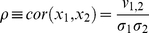
(4)is the correlation of 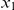 and 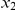 and 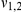 is the covariance. The variable number of kernels allowed the generation of maps with any confluence in the range 0–100%. Each synthetic map was projected onto the surface of a 3D sphere. This three-dimensional volume was sampled at a resolution comparable to confocal microscope imaging. Points located between the surfaces of the artificial microcarrier and the 3D maps were labelled as cell objects. Segmented cell regions in artificial datasets were convoluted with a Gaussian point spread function (PSF) with standard deviation estimated from real confocal datasets. This processed blurred the true signal most predominantly along the Z axis. Finally, noise was added to the whole data volume. The modelled noise had a distribution identical to the noise measured from real confocal datasets.

### Principal Component Analysis

The intensity co-occurrence matrix of the cell distribution maps was used for texture quantification, by computing contrast, correlation, energy and homogeneity [34], 35. These measurements were subjected to principal component analysis (PCA) and the first two principal components (PC) were used to monitor the morphology of the cell distributions quantitatively over time. The distribution of points in the PC space provided an instant non-subjective representation of the cell morphologies at the surface of the microcarriers. The most representative maps were identified as those located closest to the centre of the PC distributions.

## Results

### Generation of cell distribution maps

**Figure 1:**
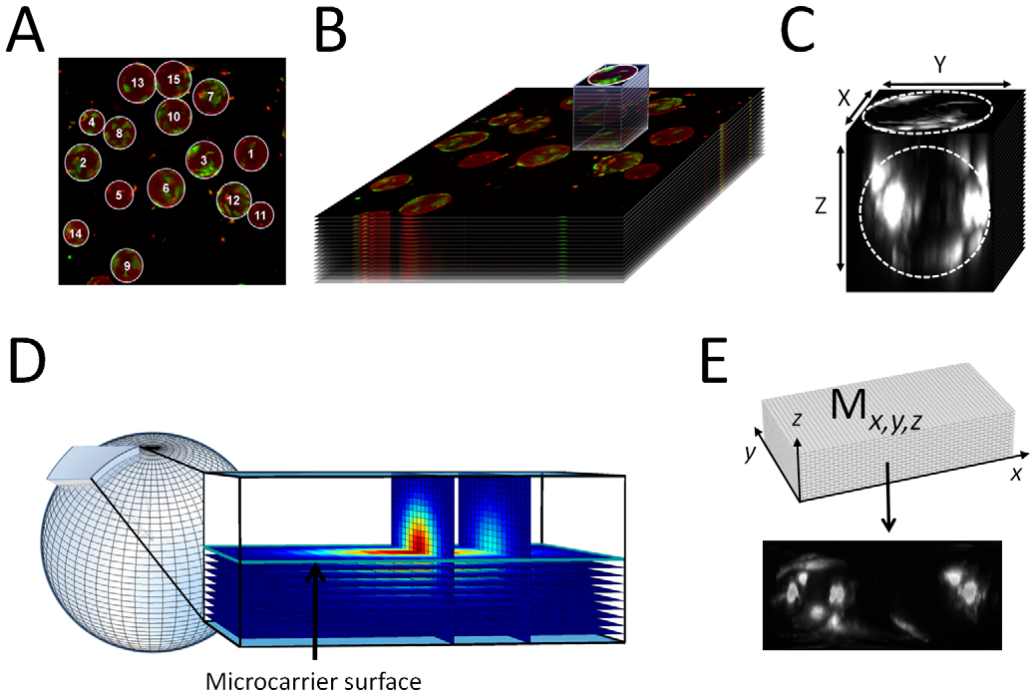
Mapping the distribution of HDF cells on the surface of microcarrier beads. *(A)* Maximum intensity projection from confocal image Z-stack with microcarriers identified by Hough transform (circles). *(B)* Extraction of sub-volume from Z-stack containing 3D fluorescence associated with a single microcarrier. *(C)* Top and side projection images calculated from sub-volume used to locate X-Y-Z coordinates of microcarrier (dashed circles and arrows). *(D)* Iterative fluorescence intensity measurements in the vicinity of the microcarrier surface (sphere) using 30 sampling spherical grids (horizontal planes in magnified sampling volume, extended to the whole microcarrier surface). *(E)* Cell distribution map (bottom) computed from unwrapped stack of sampling grids (top, M*_x,y,z_*).

Measuring the properties of cells adhered to the surface of microcarrier beads is more complex than performing the measurements in standard 2D culture due to the spatial distribution of the cells around the bead. To address this we developed an image analysis approach to process the 3D data obtained using confocal microscopy to produce 2D “cell distribution maps” which could be used for automated measurements of cell number, confluency and morphology. A summary of the process is shown in Figure 1. Confocal microscopy images of cells adhered to the surface of microcarriers were processed by applying a Hough transform-based algorithm to locate the microcarriers in the *X-Y* plane (Fig. 1A, number circles). This commonly used image analysis tool allowed individual microcarriers to be identified and the image datasets for each microcarrier to be isolated and processed independently (Fig. 1B). The confocal image stack for each individual microcarrier were then extracted into a sub-volume and processed along the *Z*-axis to identify the top and bottom coordinates of the microcarrier (Fig. 1C), which in combination with the *X-Y* data extracted previously allowed the whole microcarrier surface to be identified. Next a grid was mapped onto the surface of the microcarrier and the fluorescent intensity was measured in each grid at 30 points orthogonal to the microcarrier surface (Fig. 1D). The intensity measurements encompassed the area above and below the microcarrier surface to ensure they captured the volume of the cells adhered to the microcarriers. The gridded fluorescent intensity measurements were then unwrapped from the microcarrier sphere to create flat matrices which could be processed to create a ‘cell distribution map’ showing the location and morphology of the cells (Fig. 1E). This 2D map of the distribution of the cells on the 3D microsphere could then be used for analysis of cell number and confluency.

### Measurement of cell number and confluency

**Figure 2:**
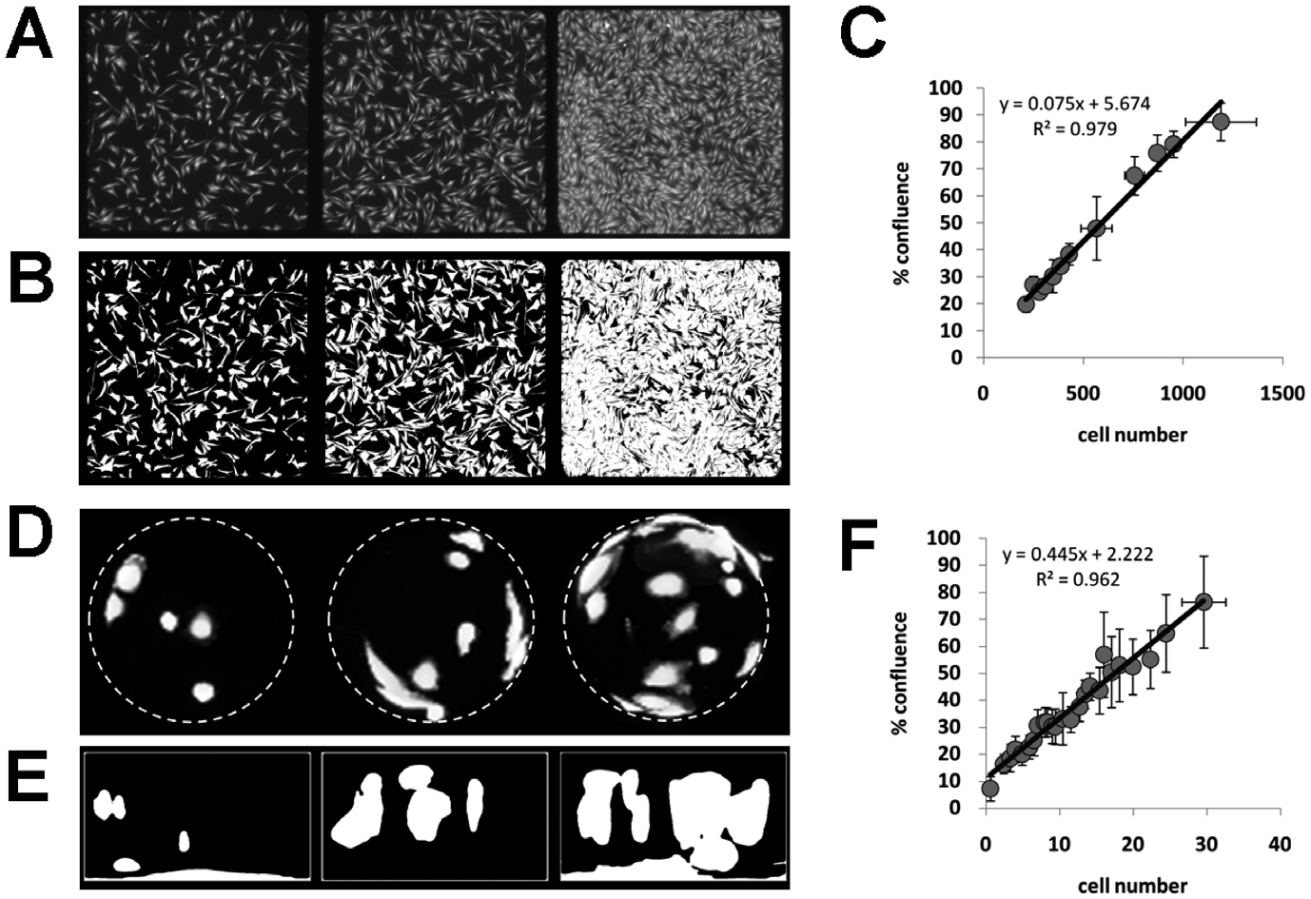
Comparison of confluency and cell number measurements in 2D and 3D culture. (A) 2D monolayer culture of fluorescently labelled HDF cells seeded on 2 mm^2^ gridded slides at three different seeding densities. (B) Cell segmentation of the images using manual thresholding to identify the individual cells. (C) Graph showing the linear relationship between cell confluency and cell number in 2D culture. (D) 3D microcarriers seeded with fluorescently labelled HDF cells at three different densities. Dashed lines show circumference of the microcarrier beads. (E) Segmented cell distribution maps processed using the cell confluency algorithm. (F) Graph showing the linear relationship between cell number and confluency for cells grown on microcarrier beads and analysed using the cell confluency algorithm.

Development of a method for automated analysis of cell confluency and its link to cell number was initially established using cells in 2D monolayer culture adhered to the surface of 2 mm^2^ gridded slides (Fig. 2A). Cells seeded at densities ranging from 200–1200 cells/grid were imaged and the cell areas were segmented using manual thresholding, which identified the cells by their fluorescence intensity compared to the background (Fig. 2B). The total surface area covered by the cells within the grids was measured and compared to manual cell number counts for each image and were shown to have a linear relationship across the cell density range (Fig. 2C). To establish if the same principle could be applied to cell attached to microcarriers, a ‘cell confluency algorithm’ was developed which could automatically segment cells using the same thresholding process applied to the cells in the 2D monolayer culture. A series of cell distribution maps were then generated from cells attached to beads at densities from <5 cells/bead (low confluency) to >30 cells/bead (high confluency) (Fig. 2D–E). Comparison of confluency measurements generated using the ‘cell confluency algorithm’ with manual cell counts from each bead also showed a linear relationship across the seeding density range (Figure 2F).

### Validation of the cell distribution mapping approach

To demonstrate that the procedure of creating cell distribution maps provides an accurate means to measure the confluency and number of cell adhered to microcarriers using the cell confluency algorithm the process was validated in two stages.

**Figure 3:**
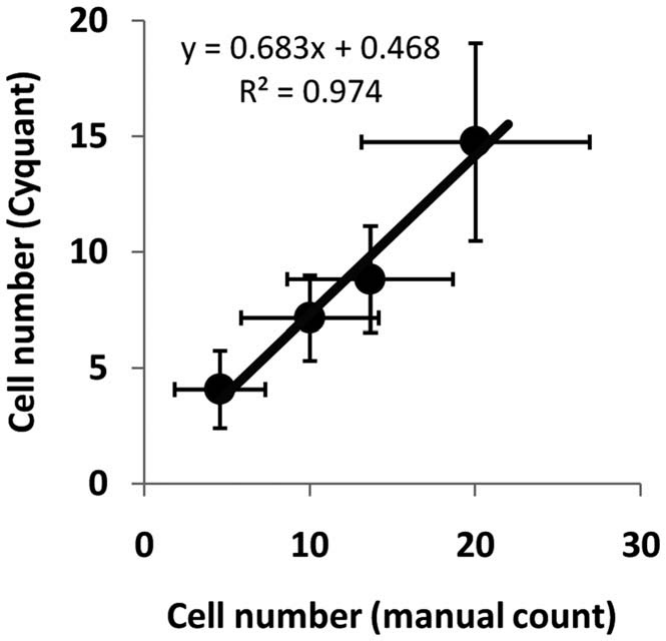
Comparison of cell number measurments. Graph to the show the linear relationship between cell number measurements obtained using the cell distribution mapping process and the commercial Cyquant assay.

In the first stage of the validation process cells were seeded onto microcarrier at 4 different densities. Samples from each density range were then either fluorescently labelled and imaged to create cell distribution maps or lysed to extract DNA for cell number analysis using the commercially available Cyquant assay. Cell numbers attained using the two processes were then compared (Fig. 3). This analysis showed a strong linear relationship (R^2^ = 0.974) between the two measurements indicating that the mapping process provides good platform for quantifying cell number.

**Figure 4:**
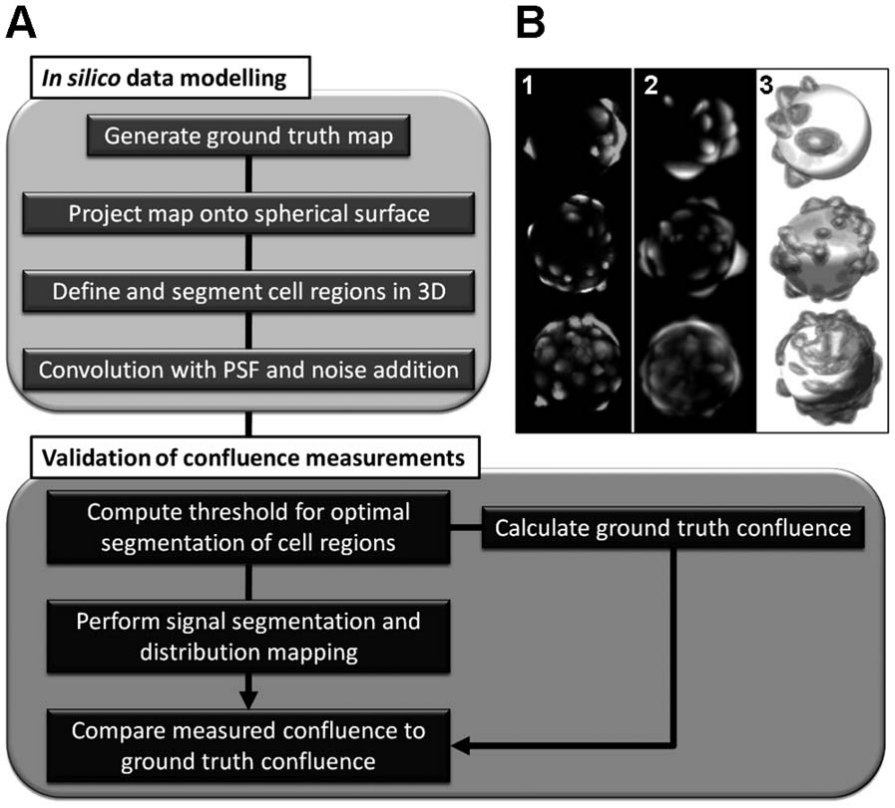
Validation of distribution mapping process with *in silico* modelling of cells adhered to microcarrier beads. *(A)* Flow diagram for *in silico* modelling (top compartment) and validation of confluence measurement (bottom compartment). *(B) 1*- Maximum intensity top projection confocal images of real HDF cells adhered to microcarrier beads. *2* – Synthetic maximum intensity top projections of cells distributions around microcarrier beads generated by *in silico* modelling. *3* – 3D rendering of the synthetic cell distribution in B2 to show cell localisation and comparability to real image data.

In the second stage of the validation process the cell confluency algorithm was used to measure the confluency of *in silico* generated cell distribution maps. This process allows a direct comparison of the confluency measurements generated by the algorithm against the known ‘ground truth’ confluency measurements from the *in silico* maps. The process also allows controlled levels of noise and point spread function (PSF) to be added to the artificial maps to measure how these imaging artefacts bias the confluency measurements. The process-map for the development of the *in silico* cell distribution maps is shown in Fig. 4A. Over 1600 *in silico* ground truth maps of cells distributions at different confluency levels were generated and projected onto spherical surfaces with the same geometric properties as the microcarrier beads. Fig. 4B shows a comparison of ‘real’ cells adhered to the surface of the microcarriers at 3 different densities (Fig. 4B “1”) compared to the *in silico* generated images of cells on beads at comparable densities (Fig. 4B “2”) and the 3D renderings of the *in silico* cells on beads (Fig. 4B “3”).

**Figure 5:**
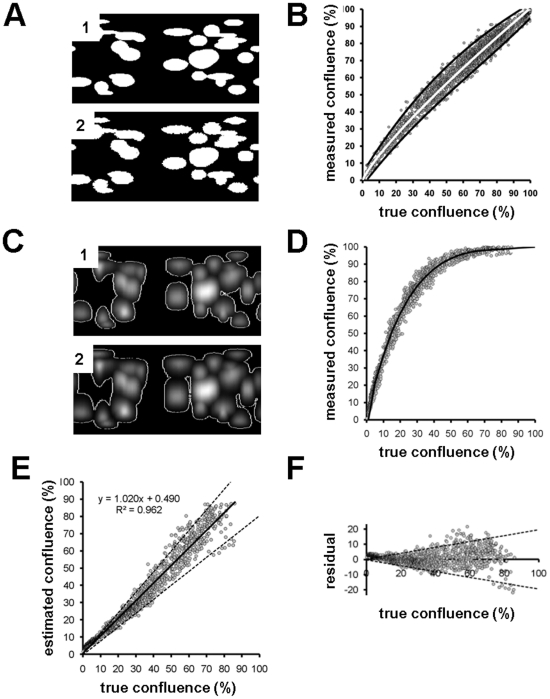
Validation of confluency measurements using cell distribution mapping. *(A)* 1 - Ground truth synthetic cell distribution map generated in absence of noise or PSF. 2 – cell confluency map generated from the synthetic data using the distribution map image processing algorithm. *(B)* Comparison of synthetic ground truth confluence versus measured confluence. The ground truth confluence is known before artificial 3D modelling (from A1) whereas the measured confluence is calculated from the map obtained after processing of the 3D model (from A2). White line = power model fit, black lines are 95% confidence intervals. *(C)* 1 - Optimal thresholding (white lines) of cell distribution map shown in A1 after convolution of the 3D model with PSF and the addition of noise. 2- Cell confluency analysis of image B1 generated using the cell distribution mapping algorithm. *(D)* Comparison of true confluence (from ground truth data) with confluence measured by the cell distribution mapping algorithm. Over-estimation from the proposed method is evident from the shape of the data distribution. However this bias can be accurately modelled (black curve). *(E)* True confluence (from ground truth maps) versus measured confluence calculated from D (*y* axis) and the fitted model (D, black curve). Estimated confluence through bias compensation restores the expected linearity between true and estimated confluences (*R* ^2^ = 0.96). Bold and dashed lines represent linear fit ±95% confidence interval. *(F)* Error estimation by residual analysis. Residual values are obtained by subtracting linear fit values from estimated confluences in E. Dashed lines represent ±95% confidence interval.

The accuracy of the cell confluency algorithm was initially validated on the *in silico* cell distribution maps in the absence of noise and PSF distortion. Fig. 5A shows an *in silico* ground truth cell distribution map (Fig. 5A “1”) and the comparable cell identification achieved using the cell confluency algorithm (Fig. 5A “2”). Comparison of the confluency measurements achieved using the cell confluency algorithm against the known ground truth measurements from *in silico* maps with 0–100% cell confluency (Fig. 5B) showed a linear response between the two measurements (*R* ^2^ = 0.982, slope = 0.92 for linear regression), indicating that in ‘clean’ images the algorithm is measuring cell confluency levels with a good degree of accuracy.

To further validate the mapping process, the *in silico* ground truth cell distribution maps underwent convolution with PSF and addition of noise to closely mimic the images obtained in real datasets. Fig. 5C shows the identification of cells for confluency measurements on an *in silico* ground truth map using optimal thresholding (Fig. 5C “1”) and the comparable cell identification using the cell confluency algorithm (Fig. 5C “2”). Comparison of these measurements from 0–100% confluency (Fig. 5D) showed a strong non-linear response between actual and measured confluences. This bias was predominantly a consequence of the PSF, which could be modelled (Fig. 5D black line) and corrected. Comparing the bias-corrected confluency measurements to the original *in silico* ground truth measurements (Fig. 5E) restored the linearity between the two measurements (R^2^ = 0.96). Residual analysis was used to calculate that the error associated with measurements of confluency using the cell confluency algorithm is approximately 10% (Fig. 5F).

### Application of image analysis for bioreactor monitoring

**Figure 6:**
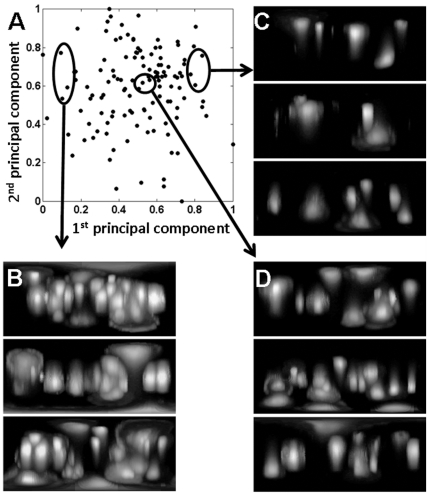
Principal Component Analysis (PCA) of the cell distribution maps. (A) Texture measurements from microcarrier seeded with 10 cells per bead and incubated for 2.5 hours were subjected to PCA and 1^st^ and 2^nd^ principal components were plotted. The PCA scatter plot provides a snapshot graphical representation of the distribution of cell morphologies. (B) Cell distribution maps taken from the left of the PCA space have the highest cell confluency. (C) Distribution maps from the right of the PCA have the lowest cell confluency. (D) The most representative cell distribution maps for the analysis are located in the centre of the PCA space.

To demonstrate that the cell distribution mapping approach could be used for measuring the confluency of live cells grown under different manufacturing conditions human dermal fibroblasts were grown at 2 seeding densities on the surface of microcarriers (5 or 10 cells per microcarrier) in stirred tank bioreactors for 13 days. Samples taken from the bioreactors at various time points were imaged, used to create cell distribution maps and analysed for cell number and confluency. The texture parameters of the maps were also subjected to principal component analysis (PCA) and the first two principal components (PC) were used to quantitatively monitor change in morphology of the cell distributions over time (Fig. 6). The distribution of points in the PC space provides a non-subjective representation of the cell morphologies at the surface of all of the sampled microcarriers (Fig. 6A). Microcarriers containing the highest cell densities are clustered on the left of the PC space (Fig. 6B), those containing the lowest cell number being clustered on the right (Fig. 6C) and the most representative maps of average cell confluency being located closest to the centre of the PC distributions (Fig. 6D).

**Figure 7:**
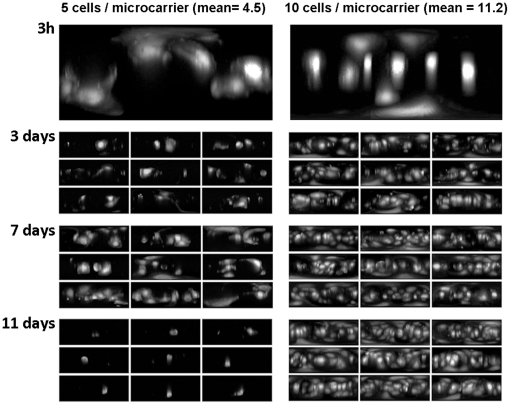
Cell distribution maps to visualise changes in cell confluency in response to cell seeding density. Microcarriers were seeded with either 5 cells per bead (left column) or 10 cells per bead (right column) and the most representative cell distribution maps from the centre of the PCA space were used to visualise difference in cell confluency over an 11 day culture period.

The most representative maps of cell growth identified from the PCA at different time points for microcarriers seeded at the two different densities are shown in Fig. 7. Uniform seeding of the cells onto the microcarrier was achieved after 3 hours of bioreactor culture for both seeding densities with an average of 4.2 cells per bead for the lower seeding density and 11.2 cells per bead for the higher density. At the lower seeding density, the cells spread more quickly onto the microcarriers, although morphologically they were not different to cells at the higher density. After 3 days however, the majority of cells became fragmented, leaving on average 2 to 3 cells per bead. Some expansion was achieved by the end of the first week, although significant bead-to-bead variability was still observed and this eventually led to majority of cells dying within the bioreactor and very few beads containing viable cells by day 13. In comparison, the microcarriers which were seeded at the higher density had numerous cell colonies or clusters within 3 days of culture, which subsequently sustained cell proliferation to the point where the majority of the surface of the microcarriers was covered by cells after 13 days in the bioreactor.

### Quantitative analysis of bioreactor cultures

**Figure 8:**
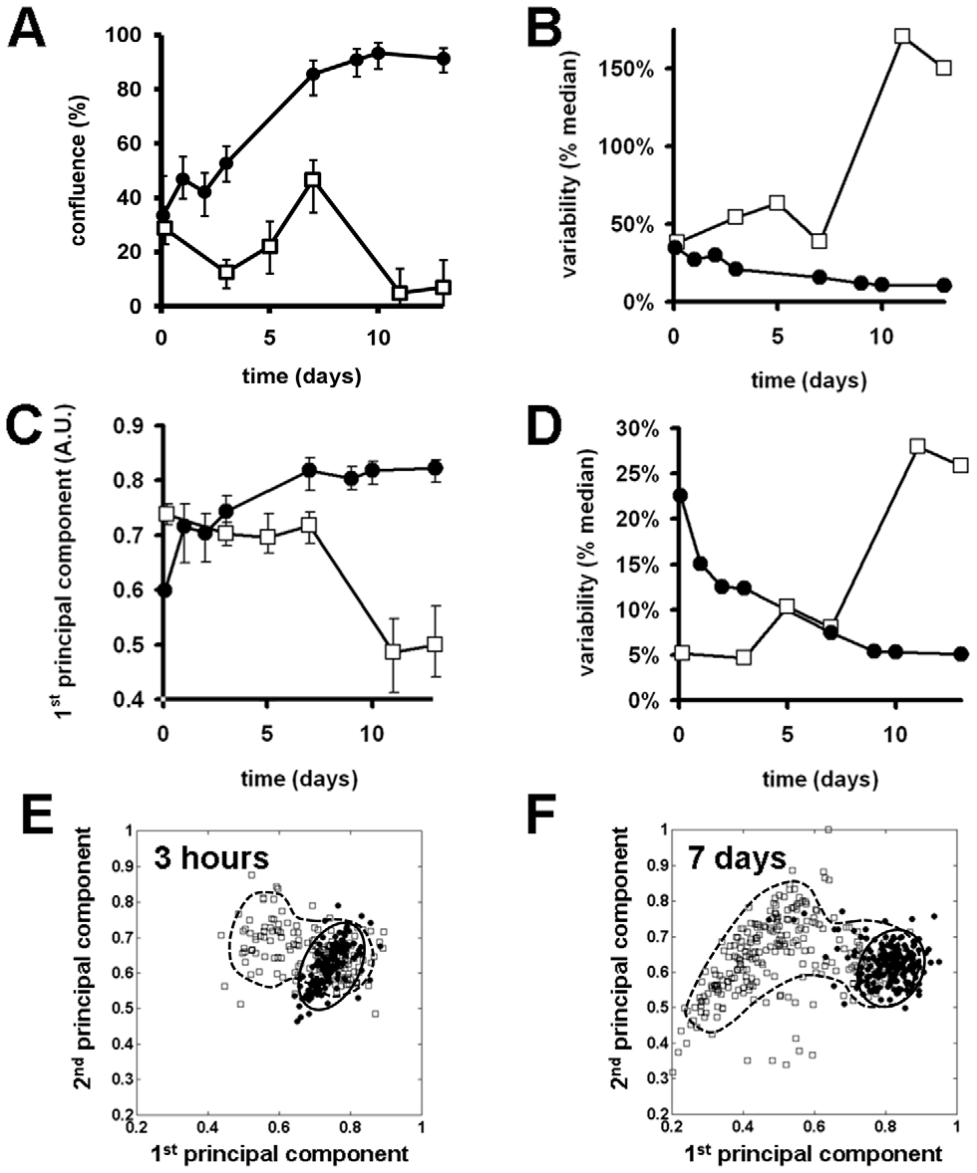
The use of PCA to quantitatively measure cell number, morphology and confluency during cell manufacture. (A) changes in cell confluency over 11 days in bioreactor culture for HDF cells seeded onto microcarriers at a density of 10 cells per bead (black squares) or 5 cells per bead (open squares). Fitted curves = median values, error bars = 16^th^–84^th^ percentiles (percentiles are used to accommodate skewed distributions. If the data were Normally distributed, the 16^th^–84^th^ percentiles would correspond to ± 1SD). (B) Measurement of bead to bead variability in cell confluency during the 11 day manufacture procedure for cells seeded onto microcarriers at a density of 10 cells per bead (black squares) or 5 cells per bead (open squares). (C) Analysis of cell morphology using the 1^st^ PC shows that microcarriers seeded at 10 cells per bead (black squares) maintain a stable morphology compared to microcarrier seeded at 5 cells per bead (open squares). (D) Analysis of the bead to bead variability in cell morphology for microcarrier seeded at 10 cells per bead (black squares) and 5 cells per bead (open squares). (E–F) The use of PCA to show differences in morphological distribution of cells seeded onto microcarrier at 10 cells per bead (black squares) or 5 cells per bead (open squares) after 2.5 hours (E) and 7 days (F) in bioreactor culture.

To quantitatively analyse the growth of cells in the bioreactor culture a series of cell distribution maps were created from samples taken over the 13 day growth period. These maps were then analysed using the cell confluency algorithm. (Fig. 8). This analysis shows that cells seeded onto microcarriers at a density of 10 cells per bead (Fig. 8A black circles) undergo an initial lag in growth over the first 3 days of culture before entering a linear growth phase and reaching almost full confluency after 7 days. In comparison, cells seeded at the lower density (Fig. 8A open boxes) underwent a decrease in confluency over the first 3 days of culture before entering a proliferative state up to day 7 and then dying within the bioreactor, such that even after 11 days of culture the average confluency of cells on the microcarriers was only ∼8%. Analysis of the confluency variability over time, defined as standard deviation divided by the median (Fig. 8B), showed that the initial variability in the confluency levels for the two seeding densities were almost the same (∼40%). However, as cells were grown in the bioreactor over 13 days the variability in bead-to-bead confluency levels for the cells seeded at the lower density significantly increased to >150% (Fig. 8B open squares) while the variability in bead confluency levels for the cells at the higher density slowly decreased to <10%. This low inter-bead variability at the higher density demonstrates that cell growth is progressing evenly throughout the bioreactor culture.

In addition to confluency measurements, cell morphology could also be quantified using PCA to give a measure of cell health within the bioreactor environment (Fig. 8C–D). This analysis shows that cells seeded at the higher densities initially have a larger cell to cell morphological variability (∼22%) than cells at the lower seeding density (∼5%). However, morphological variability in the higher density culture then decreases over the first 7 days as the cells are proliferating eventually reaching a plateau as the cells become fully confluent (∼5%). In comparison morphological variability in the lower seeding density culture is stable for the first 7 days even though the cells are proliferating. As the cells begin to die after 7 days of culture the morphological variability dramatically increases (∼28%) indicating an unhealthy culture.

PCA can also be used to graphically compare the morphologies between low and high seeding densities (Fig. 8E–F). At the time of seeding the cells onto the beads the PCA analysis shows that the morphology of the cells at the higher density forms a tight cluster on the right-hand side of the PCA space (Fig. 8E black line) while the cells seeded at the lower density form a less compact but overlapping cluster (Fig. 8E dashed line). By 7 days of culture which correspond to the time immediately before the cells at the lower seeding density will begin to die, the PCA analysis shows a clear divergence in cell morphologies between the two culture conditions. The higher seeding density cells are still tightly clustered in the same location with the PCA space (Fig. 8F black line). However, the lower seeding density cells have strongly shifted with the PCA space forming a large distinct cluster on the left hand side (Fig. 8F dashed line), which in this case indicates a cell morphology associated with an unhealthy bioreactor culture.

## Discussion

As regenerative medicine products transition from the laboratory into clinical application the requirement to produce cells in commercially viable quantities is driving the use of bioreactor based manufacturing [1]. This approach presents a number challenges particularly as many of the cell models used for therapeutic application require adherence to a solid substrate on which to grow. In turn, this makes it difficult to perform biological measurements directly on the cells to ensure optimal growth and allow quality control of the production process [31].

In standard 2D cell culture, batch quality control throughout the cell expansion process generally includes subjective measurements of cell confluency and cell morphology as well as direct measurements of cell number. However, there are currently no methods to measure all these parameters simultaneously in 3D bioreactor culture systems. Instead, bioreactor measurements tend to analyse a single property such as cell number. This provides only limited information and does not provide a quantitative representation of important parameters such as the homogeneity of the cell population or the morphology and distribution of the cells. In this paper, we have described a versatile yet powerful method to achieve multiplexed measurements of cell number, confluency and morphology in 3D bioreactor cultures. This is achieved by processing the data generated from confocal microscopy imaging to create a 2D map of the distribution of the cells on their 3D growth substrate while maintaining the cell morphology information. The cell distribution maps can then be used for automated analysis of cell number and confluency using routine segmentation and thresholding approaches. This method was designed to incorporate commonly used procedures such as bioreactor sampling, fluorescent cell labelling and microscopy imaging with a series of validated algorithms which are operated using the commercially available Matlab platform in order to allow the procedure to be easily used in other laboratories. The analysis time for this method is comparable to cell counting methods employed in standard 2D culture which, depending on the assay used, can range from a few minutes (e.g. trypan blue assay) to several hours (e.g. MTT assay). In this study, the fluorescent Calcein-AM labelling of viable cells has an assay time of less than 30 minutes, allowing the cells to be labelled without affecting viability and morphology. On a typical confocal imaging platform, another 30 minutes are necessary to acquire the raw image datasets to constitute a statistically relevant sample. Once the raw data is acquired, image processing and analysis can be performed in approximately 30 minutes giving a total assay time of about 1.5 hours. However, this could be improved through parallel processing (simultaneous to data acquisition), which would potentially ensure that the whole process, from cell labelling to quantitative analysis, would take just 60 minutes. The image processing and analysis software has also been thoroughly validated using fully characterised *in-silico* 3D models replicating real datasets and has been shown to have an accuracy of 90% which is comparable to standard cell number assays such as MTT.

The use of cells in therapeutic applications requires stringent monitoring during the manufacture process. This makes it important to be able to link measurements such as cell number to the health and quality of the cells during processing. The approach undertaken in this study presents a number of advantages for use in quality control procedures. Firstly, only small samples are required to obtain statistically relevant data, in this study a 5 ml sample was used for labelling and microscopy but, dependant on the concentration of microcarriers in the bioreactor, this could be reduced significantly. For example, the bead concentration presented here was approximately 52,000 beads/ml which would mean that >200 beads could be analysed from a bioreactor sample as small as 5 µl. This small sampling size is particularly attractive for the manufacture of cell therapy products where a significant proportion of the product cost is tied up in the manufacture of the cells [36]. The use of this imaging approach with fluorescent dyes such as Calcein-AM also offers the potential for the samples to be fixed post-labelling and stored at 4°C for several weeks permitting subsequent detailed analysis if, for example, end-of-process cells are not of sufficient quality. Storage of raw images and processed maps also offers the possibility of data re-analysis, currently not achievable when only a simple cell count is performed or few illustrative micrographs are taken. Importantly, the analysis of the data itself is fully quantitative and does not rely upon subjective assessment by trained staff, thereby allowing traceability throughout the manufacture process.

The transition from standard cell culture to bioreactor based manufacture is driven by practical issues associated with handling large cell volumes and economic decisions regarding product cost reduction. For human cells used in cell therapy products, which are typically slow to expand in culture, it is important to have methods in place which can be used to reduce manufacture costs by optimising processes and identifying problems quickly [37]. The cell distribution mapping approach described here, in combination with quantitative analysis of cell number and confluency go someway to helping achieve this. In this study we show how cells seeded at an initial density of 10 cells per bead have reached maximum confluency within 7 days of bioreactor culture, while cells seeded at 5 cells per bead have only reached 44% confluency during this time. Even though under these conditions the lower seeding density cells eventually died, if they had a maintained a linear proliferate rate it would have taken approximately 7 more days of culture for them to reach full confluency. Having this type of quantitative information to hand can therefore be invaluable in helping inform the decisions making processes about the economic trade of between initial seeding densities and the length of manufacture time.

Image based morphological measurements of individual cells in 2D systems is the principle behind powerful platform technologies such as high content screening (HCS). These technologies are being used to ‘industrialise’ cell analysis particularly in drug discovery research by combining automation, sample preparation, image acquisition, processing and analysis to permit subtle changes in cell morphology be measured in great detail [38]–[40]. The principals of HCS are equally applicable for monitoring the health of cells during bioreactor manufacture but have not yet been adapted for this purpose. With therapeutic cell lines, changes in cell morphology can be indicative of a problem with the manufacture process which in turn may have detrimental effects on the quality of the cells (e.g. reduced expansion by increased contact inhibition or spontaneous differentiation of stem and progenitor cells). In this study we used well described texture measurements [35], [41] to quantify and classify cell morphology on the microcarriers. Under different manufacturing conditions, such as cell seeding density, PCA clearly identified two morphologically distinct distributions amongst the cell populations. One of which does not lead to cell expansion. This approach could therefore be used to identify sub-optimal cultures rapidly following seeding preventing wasted manufacturing time. The mapping process described in this study also considerably compressed the information contained in the original 3D image volumes, in this case reducing the size of the dataset for a statistically relevant analysis by a factor of 43. However, under optimal conditions this compression factor could be increased to >200 without significant loss of information. This dramatically increases the processing speed for image data analysis overcoming some of the data management bottlenecks that have been encountered with HCS [42]. Furthermore, as cell technologies change, and in particular as 3D culture technologies become incorporated into drug screening programmes, the type of analysis system described in this paper could also support the transition of HCS into high-throughput 3D drug development cell models [43].

Although the approach presented in this study has been shown to be robust and reliable, there is potential for improving the image analysis software by incorporating deconvolution algorithms or introducing different texture metrics that are specific for a particular cell type. This would be useful for cell therapy products based on stem cells or other progenitor populations which incorporate a differentiation step following cell expansion. A good example of this would be monitoring the production of neural cells for the clinical treatment of neurodegenerative diseases. It has been shown that morphological measurements can be used to assess the differentiation of stem cells along a neuronal lineage identifying, for example, the development of neurite outgrowths typical of developing neurons [44]. This could also be combined with fluorescent immunocytochemistry to incorporate morphology measurements alongside cell specific markers such as β-III tubulin, GFAP and O4 to identify developing subpopulations of neurons, astrocytes and oligodendrocytes during the manufacture process.

In conclusion, as cell manufacture transitions from standard culture into bioreactor based processing the tools to monitor cell growth and measure cell quality need to be in place. The cell distribution mapping approach described in this paper goes some way to achieving this by providing a system for the unbiased multiplexed measurement of cell number, confluency and morphology. This offers distinct advantages over current methods of analysis, which typically measure a single parameter, by providing a more comprehensive evaluation of cell growth and allowing bioreactor culture performance to be optimised. In addition the minimal number of manual and sample handling steps combined with fast automated analysis would facilitate the use of this type of analytical approach within a cell manufacture facility and could help in the transition towards bioreactor based manufacture for clinical grade cells.
